# Heart and Brain Responses to Real Versus Simulated Chess Games in Trained Chess Players: A Quantitative EEG and HRV Study

**DOI:** 10.3390/ijerph16245021

**Published:** 2019-12-10

**Authors:** Juan Pedro Fuentes-García, Telmo Pereira, Maria António Castro, António Carvalho Santos, Santos Villafaina

**Affiliations:** 1 Faculty of Sport Science, University of Extremadura, Avda: Universidad S/N, 10003 Cáceres, Spain; jpfuent@unex.es; 2 Polytechnic Institute of Coimbra, Coimbra Health School, 3046-854 Coimbra, Portugal; telmo@estescoimbra.pt (T.P.); mac@estescoimbra.pt (M.A.C.); acs@estescoimbra.pt (A.C.S.); 3 Centre for Mechanical and Engineering Materials and Processes, University of Coimbra, 3030-788 Coimbra, Portugal

**Keywords:** chess, brain, attention, virtual reality, EEG, autonomic modulation

## Abstract

The aim of the present study was to investigate how the heart and the brain react to playing chess with a computer versus in a real context in chess players. We also aim to investigate if familiarization with simulated practice leads to changes in heart rate variability (HRV) and the electroencephalographic (EEG) power spectrum. We designed a cross-sectional study, enrolling 27 chess players. They were randomly assigned to 3 minutes plus 2-second chess games: one with a computer (simulated scenario), and another in a real context. Additionally, participants were divided into two groups according to their level of familiarization of playing chess in a computer context. While they were playing, HRV and EEG were continuously recorded. Differences in HRV and EEG theta power spectrum between playing chess in a real or a simulated scenario were not found in chess players (*p*-value > 0.05). When participants were divided into groups (familiarized and unfamiliarized with simulated chess practice), significant differences were observed in HRV and EEG (*p*-value < 0.05). The EEG theta power spectrum was significantly lower, and HRV was higher in unfamiliarized players during the simulated scenario, which could indicate that they were less focused in a simulated environment than in a real context. Therefore, familiarization with simulated environments should be taken into account during the training process to achieve the best performance.

## 1. Introduction

The game of chess has been useful in the study of basic cognitive processes such as memory, decision making, or problem solving [1,2]. Moreover, chess practice could improve mathematical problem-solving ability or logic [3,4] due to the cognitive processes involved in this game [3,4]. In the last few decades, due to technology development, chess has been experiencing a change in the methodological models of practice. Several engines and webpages have appeared, allowing people to play chess without a physical board or pieces in a simulated environment.

Encephalogram (EEG) has been commonly used to study cognitive processes. In this regard, changes in theta power spectrum (4–8 Hz) have been detected during focused attention when the difficulty of a task increases [5,6] or with emotional responses such as stress [7,8]. Moreover, the heart rate variability (HRV) has been considered as a measure of heart–brain interaction [9], and it is also modified by cognitive, attentional tasks or anxiogenic response [10,11,12,13]. Both EEG and HRV are highly applicable tools to control and monitor workload in chess players [14,15]. Due to the emergence of virtual reality and simulated contexts, the study of the quality of these environments is of great interest for researchers and clinicians. Similar to this paradigm, a previous study has compared the heart rate as well as the EEG response in a simulator vs. real drive [16]. Moreover, previous studies in aviation showed that the psychophysiological response is very analogous in the simulator and in real flight [17,18,19]. In this regard, if compared to the psychophysiological response of chess players while playing chess in a computer vs. a real environment, differences should not be expected.

However, to the best of our knowledge, no previous research addressed the differences between playing chess with computer or in a real environment (with a real board). Thus, the aim of the present study is to analyze the HRV and the EEG power spectrum in chess players playing with a computer and on a board. In addition, we also aim to investigate the influence of chess playing familiarization with computers in HRV and EEG power spectrum.

## 2. Materials and Methods

### 2.1. Participants

A total of 27 chess players were enrolled in a cross-sectional study. The mean age was 22.69 (11.87) years old with a chess playing experience of 9.20 (4.34) years. This sample size, with two observations per subject, achieved 100% power to detect significant differences (*p*-value < 0.05) using the RMSSD values of the present study. The PASS software for performing power and sample size calculations (version 11.0; PASS; Kaysville, UT, USA) was employed.

Elo rating system was developed by Elo [20]. This index indicated the chess playing level and it is calculated by competitor-versus-competitor games (the higher the ELO, the higher the level of the chess player) [21]. This rating system is used by the Federation Internationale des Echecs (FIDE). Participants had a mean ELO of 1661 (337.15). In the present study, chess players were classified in two group depending to their familiarization with playing in a simulated environment (computer). A visual analog scale (0–10) was used to ask for the familiarization with playing chess with computer. If the score was ≤5, we considered that the chess player was not familiarized with this environment. The familiarized chess players (*n* = 14) had a mean age of 25.08 (12.70) and an ELO ranged between 1267 and 2278. The unfamiliarized with computer chess players (*n* = 13) had a mean age of 20.11 (10.81) and an ELO ranged between 1076 and 2200.

None of the participants were under medication that could affect the autonomic nervous system. Moreover, they gave written informed consent to participate in the study, and according with the Declaration of Helsinki, the procedures were approved by the University Research Ethics committee (approval number: 85/2015). Exclusion criteria included: (1) inability to perform the tasks with the computer or chess board; (2) diseases that affect the autonomic nervous system; and (3) not being classified by the International Chess Federation with ELO.

### 2.2. Procedure

Before starting the study, participants were informed about the procedures and protocol requirements during the chess games. Thus, all of them underwent five minutes of familiarization period with both the chess board and the computer used in the study. The experimental room was calm, and light and temperature were continuously regulated. All the chess games were performed in the afternoon.

Participants conducted randomly two chess games: (1) 3 minutes plus 2 seconds using a computer environment and (2) 3 minutes plus 2 seconds using a real board environment. While moves in the computer environment were done immediately, in the real board environment, a trained technician immediately transferred the moves, which the chess engine indicated, into the chess board.

To respond to the participants moves, a Fritz 15 software (ChessBase GmbH, Hamburg, Germany) was used, using Stockfish 6, 64 BIT, as chess engine (GPL3 licence- General Public License Version 3). The Stockfish 6 is one of the strongest chess engines in the world and it is open source and is commonly used as a tool for chess training due to the similarities with the tactical responses given by humans [22]. ASUS laptop was employed (Intel Core i7-6500U, 1 TB, 8 GB memory DDR3L-SDRAM). One researcher selected the Fritz level according to the ELO level of each player.

### 2.3. Instruments

The EEG and the electrocardiogram (ECG) data were registered using an MP150 platform with EEG100C and ECG100C amplifiers, respectively, running through the AcqKnowledge 4.4 software (BIOPAC^®^ Systems, Inc. USA). The EEG data was filtered using a 0.5 Hz high-pass filter and a 35 Hz low-pass notch filter, through which a 50 Hz notch filter was in place. Impedances were checked using the electrode impedance checker (EL-CHECK, BIOPAC^®^ Systems, Inc. USA), and kept below 5 kΩ. The ECG was recorded following the recommendation of the Task Force of the European Society of Cardiology and the North American Society of Pacing and Electrophysiology [23]. A DII-like lead was composed by placing two Ag/AgCl electrodes: one on the left leg (positive) and other on the right arm (negative), with a third Ag/AgCl electrode (ground) being placed over the right leg. An acquisition sample rate of 1000 Hz was used, and recordings were made in the NORM mode through a 35 Hz low-pass notch filter with a 50 Hz notch filter, and a 0.5 Hz high-pass filter.

### 2.4. Outcomes

EEG was recorded from 13 scalp locations according to the International 10–20 system: frontal (Fz, F3, and F4), central (Cz, C3, and C4), temporal (T3 and T4), parietal (Pz, P3, and P4) and occipital (O1 and O2).

Ground electrodes were placed in the earlobe. A sampling rate of 250 Hz was used. For pre-processing and data analysis, the EEGlab toolbox (MatLab) was utilized [24]. After applying a 1 Hz high-pass filter, the line noise was removed. To reject bad channels and correct continuous data, the Artifact Subspace Reconstruction (ASR) was used. Then, bad channels were interpolated and the data was re-referenced to average. Independent Component Analysis (ICA) was performed [25] and single equivalent current dipoles were estimated. The symmetrically-constrained bilateral dipoles were searched. Independent components (ICs) whose dipoles’ residual variance was larger than 15% were removed as well as those with dipoles located outside the brain. Power Spectral Density were computed and banded into theta (4–7 Hz) frequency bands.

The ECG data was exported in .edf format to the Kubios HRV software (v. 2.1) (Biosignal Analysis and Medical Imaging Group, Kuopio, Finland) [26] in order to obtain time and frequency domains, as well as non-linear measures. A medium filter was applied to correct artifacts, and the correction level identified all beat to beat intervals (RR) that were longer/shorter than 0.25 seconds compared to the local average. The correction was made by replacing the identified artifacts with interpolated values using a cubic spline interpolation. Smoothness prior method with a lambda value of 500 was used to remove disturbing low-frequency baseline trend components [27].

The extracted variables were: in the time domain, mean RR interval, the standard deviation of all normal to normal RR intervals (SDNN), and the root mean square of successive differences RR interval differences (RMSSD) were analyzed. In the frequency domain, the low frequency (LF) (ms^2^), the high frequency (HF) (ms^2^), the ratio between LF/HF and the total power, were calculated. Additionally, non-linear measures such as the dispersion, standard deviation, of points perpendicular to the axis of line-of-identity in the Poincaré plot (SD1); the dispersion, standard deviation, of points along the axis of line-of-identity in the Poincaré plot (SD2); and the sample entropy (SampEn) were also included in the analysis. Higher values of SDNN, RMSSD, SD1, as well as lower values of LF/HF and SD2, are associated with higher parasympathetic influence [28,29,30,31,32].

### 2.5. Statistical Analysis

EEGLAB STUDY design was used to compare the electrophysiological response during real vs. computer environment. Moreover, participants were divided into two groups: familiarized and unfamiliarized with computer chess game environment. Thus, an EEGLAB STUDY design (2 × 2) was configured to compare familiarized with unfamiliarized with computer chess participants in the two conditions (computer vs. real environment). Non-parametric analysis (permutation analysis) was computed. In order to control the Type I error, the false discovery rate correction (FDR) was applied.

Moreover, the SPSS statistical package (version 22.0; SPSS (IBM, Armonk, NY, USA), was used to analyze the HRV data. Wilcoxon signed rank test was used to assess differences between the two conditions (real vs. computer environment), as well as to analyze sub-groups differences in the above mentioned conditions.

## 3. Results

### 3.1. Overall Comparisons between Playing with Chess Board vs. Computer

**Figure 1:**
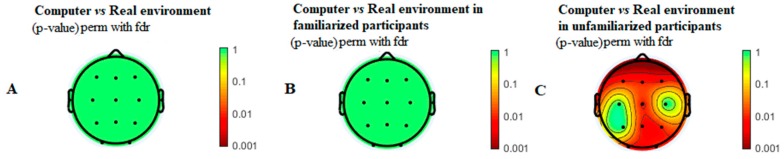
Topographic maps of the interactions in the theta power spectrum (4–7 Hz) in the different analyses. (**A**) Topographic map of the comparison between computer vs. real chess context. Differences were not found (*p* > 0.05). (**B**) Topographic map of the comparison between computer vs. real chess context in those participants who were familiarized with playing chess with computer. Differences were not found (*p* > 0.05). (**C**) Topographic map of the comparison between computer vs. real chess context in those participants who were not familiarized with playing chess with computer. Differences were located (*p* < 0.05) in F3, Fz, F4, Cz, T3, T4, Pz, P4, O1, and O2, showing higher values of theta while playing in a real chess environment.

Figure 1A shows the comparison between playing chess with computer or with chess board. Statistically significant differences between these two conditions were not found in EEG theta power spectrum.

All the analyses were performed applying a non-parametric analysis applying false discovery rate (FDR) correction as determined by paired *t*-test.

Moreover, Table 1 shows the comparison between playing chess with a computer or with a chess board in HRV. Significant differences were not found in any of the studied variables.

### 3.2. Playing with Computer vs. Chess Board in Chess Players Unfamiliarized with Playing Chess in Computer Environment

Sub-groups did not statistically differ in age, years playing chess or ELO (*p*-value > 0.05). Significant differences were found when studying the influence of playing chess with the computer or with chess board in chess players who are unfamiliarized with playing chess with computer (see Figure 1C). In this regard, theta EEG power spectrum is higher in frontal (F3, Fz, and F4), central (Cz), temporal (T3 and T4), parietal (Pz and P4), and occipital areas (O1 and O2) in chess board environments when compared with playing chess with a computer.

In the same line, Table 2 shows that HRV variables such as RMSSD or SD1 are significantly lower when playing chess with board.

### 3.3. Playing with Computer vs. Chess Board in Chess Players Familiarized with Playing Chess in Computer Environment

Sub-groups did not statistically differ in age, years playing chess, or ELO (*p*-value > 0.05). This subgroup was composed of 14 chess players. Figure 1B shows the theta EEG power spectrum, where statistically significant differences were not found. In addition, Table 3 shows the HRV analysis, where differences between playing chess with computer or with a chess board were not found.

## 4. Discussion

The present study examined the differences between playing chess in a computer environment or in a real context (against an opponent on a real board). Overall, statistically significant differences were not found when playing chess in a real environment vs. computer environment. However, when chess players were divided in two groups (familiarized and unfamiliarized with playing chess with computer), differences appeared. Unfamiliarized chess players obtained less values of EEG theta power spectrum, as well as higher values of HRV (RMSSD and SD1) when playing chess in a computer.

Previous studies have used the EEG to explore the brain electrical processing in real and simulated environment in order to compare how real the simulated environment were [16,33,34]. In this regard, in our study, generally speaking, we did not find significant differences between playing chess in a computer or in a real environment. This is in line with previous studies in aviation [17,18,19]. For example, Magnusson and Berggren [18] showed that the HRV response was very analogous in the simulator and in real flight [18]. This indicated that the pilots invested the same mental effort during the real or the simulator environment.

However, differences in EEG theta power spectrum were detected in those chess players who were unfamiliarized with playing chess with computer. Theta power has been related to focused attention as well as with task difficulty increases (increasing theta power spectrum when difficulty increases) [5,6]. The prefrontal cortex (PFC) has been described as the center of cognitive function, being involved in executive functions such as decision making and problem-solving [35,36,37]. Moreover, brain imaging studies have identified a distributed network of areas in the PFC and parietal cortex, which seem to be involved in the allocation of attention [38,39,40,41]. In this regard, our EEG results showed that differences between familiarized vs. unfamiliarized participants were predominantly located in frontal and parietal areas. These results could be relevant for the training of these chess players (unfamiliarized with playing chess with computers), indicating that the focused attention, and in consequence the quality of the training, may be higher using real environments. Since simulation environments are frequently used as training in other sports or fields [42,43,44], future studies should use the EEG or the HRV in order to test how real the simulation environments are. Moreover, the study of the effectiveness of computer-based and real environment trainings in chess players would be interesting.

Regarding the HRV analysis, results are in line with those obtained in the EEG. Although differences are not generally observed, in those chess players who were unfamiliarized with playing chess with computer, differences between real and computer environment appeared. These participants reached higher values of HRV while playing in the computer environment. In this regard, HRV is a non-invasive method for studying the balance between sympathetic and parasympathetic nervous system [9]. Previous studies have demonstrated how cognitive tasks could modulate HRV response [10,12], even during chess problems [45], where chess players obtained higher values of HRV during the easier problem-solving tasks.

Considering both EEG and HRV, and taking into account that the difficulty of the task was the same in the two planned chess games, we hypothesize that changes in the psychophysiological response could indicate that those unfamiliarized with playing chess with computer players were more focused during the real chess environment than during the chess computer environment. In this line, previous studies in the field of aviation [18,19] reported differences in the psychophysiological reactions between the simulated and the real environment. However, although the psychophysiological response between simulated and real environments was similar, authors observed that the reactions started at different levels, showing extra arousal in the real situation. This could be explained because when entering the real aircraft, pilots know they are exposed to real danger [18]. Similarly, in our study, those unfamiliarized with computer games identified the simulated environment as unreal, so attention could be attenuated. This could explain the psychophysiological response of this subgroup.

One of the possible limitations of the present study was the relatively small sample size of the subgroups. Hence, we cannot generalize the results to the general population. Another possible limitation is the fact that chess computer engines tend to respond faster than humans do. Thus, we cannot totally simulate a real environment. However, playing against the computer allowed us to exactly regulate the level of which players had to play, creating two situations (board and computer) with the same level of difficulty.

## 5. Conclusions

This is the first study which explores the differences between playing chess in a real or in a computer environment. Differences in EEG theta power spectrum and HRV were obtained only in chess players who were not used to playing chess in a computer environment. These differences could indicate that those chess players unfamiliarized with playing chess with computer were less focused in a simulated environment than in a real environment, which may have important implications for the training strategies in order to achieve the best performance in these players.

## Figures and Tables

**Table 1:** Heart rate variability comparison between computer vs. board playing (*n* = 27).

Variable	Board Median (IQR)	Computer Median (IQR)	*p*-Value	Effect Size
MeanRR	706.98 (122.48)	777.86 (136.61)	0.055	−0.370
SDNN	49.45 (55.52)	48.98 (33.77)	0.456	−0.143
pNN50	18.21 (19.10)	17.92 (24.39)	0.387	−0.166
RMSSD	38.27 (22.91)	39.94 (32.34)	0.143	−0.282
HFn	39.19 (18.00)	38.24 (28.43)	0.456	−0.143
LFn	60.66 (17.95)	61.67 (28.44)	0.471	−0.139
LF/HF	1.55 (1.41)	1.61 (2.36)	0.829	−0.004
Total Power	2123.53 (2952.96)	1914.01 (2883.11)	0.471	−0.139
SD1	27.11 (16.24)	28.28 (22.90)	0.136	−0.287
SD2	65.30 (31.45)	59.04 (37.05)	0.239	−0.227
SampEn	1.64 (0.39)	1.59 (0.57)	0.792	−0.050

Effect size correspond to “*r*”. IQR: interquartile range; RR: time between RR intervals in milliseconds; SDNN: standard deviation of all normal to normal RR intervals; pNN50: the percentage of intervals >50 ms different from preceding interval; RMSSD: root mean square of successive RR interval differences; LF/HF: ratio low frequency (LFn) (ms^2^)/high frequency (HFn) (ms^2^); Total Power: the sum of all spectra; SD1: dispersion, standard deviation, of points perpendicular to the axis of line-of-identity in the Poincaré plot; SD2: dispersion, standard deviation, of points along the axis of line-of-identity in the Poincaré plot; SampEn: sample entropy.

**Table 2:** Heart rate variability comparison between computer vs. board playing in participants who are not familiarized playing with computer (*n* = 13).

Variable	Board Median (IQR)	Computer Median (IQR)	*p*-Value	Effect Size
MeanRR	696.63 (98.56)	767.55 (132.64)	0.075	−0.494
SDNN	50.28 (24.63)	49.16 (30.49)	0.701	−0.106
pNN50	19.58 (22.74)	25.98 (21.73)	0.249	−0.320
RMSSD	42.01 (24.34)	45.04 (39.40)	0.028	−0.610
HFn	41.82 (15.17)	47.24 (33.53)	0.249	−0.320
LFn	57.90 (14.94)	51.62 (33.58)	0.279	−0.307
LF/HF	1.38 (0.89)	1.09 (2.11)	0.807	−0.184
Total Power	2496.56 (2530.04)	1914.01 (3222.14)	0.471	−0.048
SD1	29.75 (17.23)	31.88 (27.90)	0.028	−0.610
SD2	65.91 (28.67)	62.63 (32.84)	0.507	−0.184
SampEn	1.64 (0.30)	1.64 (0.60)	0.807	−0.068

Effect size correspond to “*r*”. IQR: interquartile range; RR: time between RR intervals in milliseconds; SDNN: standard deviation of all normal to normal RR intervals; pNN50: the percentage of intervals >50 ms different from preceding interval; RMSSD: root mean square of successive RR interval differences; LF/HF: ratio low frequency (LFn) (ms^2^)/high frequency (HFn) (ms^2^); Total Power: the sum of all spectra; SD1: dispersion, standard deviation, of points perpendicular to the axis of line-of-identity in the Poincaré plot; SD2: dispersion, standard deviation, of points along the axis of line-of-identity in the Poincaré plot; SampEn: sample entropy.

**Table 3:** Heart rate variability comparison between computer vs. board playing in participants who are familiarized playing with computer (*n* = 14).

Variable	Board Mean (SD)	Computer Mean (SD)	*p*-Value	Effect Size
MeanRR	712.06 (196.64)	781.07 (179.92)	0.300	−0.286
SDNN	47.58 (32.67)	44.36 (32.62)	0.397	−0.235
pNN50	11.53 (25.03)	13.38 (26.67)	0.975	−0.008
RMSSD	36.00 (25.50)	36.48 (30.79)	0.975	−0.008
HFn	33.80 (22.12)	33.93 (27.71)	0.925	−0.026
LFn	66.19 (22.27)	66.01 (27.75)	0.875	−0.043
LF/HF	2.00 (2.14)	1.94 (2.69)	0.510	−0.182
Total Power	1658.37 (3206.29)	1774.21 (2319.68)	0.638	−0.130
SD1	25.49 (20.19)	25.85 (21.87)	0.975	−0.008
SD2	59.67 (42.78)	56.72 (41.39)	0.363	−0.252
SampEn	1.68 (0.62)	1.57 (0.60)	0.826	−0.061

Effect size correspond to “*r*”. IQR: interquartile range; RR: time between RR intervals in milliseconds; SDNN: standard deviation of all normal to normal RR intervals; pNN50: the percentage of intervals >50 ms different from preceding interval; RMSSD: root mean square of successive RR interval differences; LF/HF: ratio low frequency (LFn) (ms^2^)/high frequency (HFn) (ms^2^); Total Power: the sum of all spectra; SD1: dispersion, standard deviation, of points perpendicular to the axis of line-of-identity in the Poincaré plot; SD2: dispersion, standard deviation, of points along the axis of line-of-identity in the Poincaré plot; SampEn: sample entropy.
